# Does a Dog Improve the Mental Well-being of Patients and Staff in a CAMHS Hospital?

**DOI:** 10.1192/bjo.2022.388

**Published:** 2022-06-20

**Authors:** Michael Foster, Mica Quinn

**Affiliations:** North Staffordshire Combined Healthcare CAPS Trust, Stoke-On-Trent, United Kingdom

## Abstract

**Aims:**

The aim was to create and deliver support sessions with the psychiatry consultant's dog, Rupert, to improve the emotional health of both patients and staff. It was hypothesised that having time with a calm and affectionate dog would reduce both young person and adult anxiety, improve their mood, and help them communicate. Since the start of the COVID-19 outbreak, there has been a gradual increase in demand from children and adolescent mental health services (CAMHS), and consequent pressure on NHS staff. On June 2021, Rupert was registered as an emotional support dog with the Trust and began weekly visits to the Darwin Hospital, Stoke-On-Trent. This is a 12 bedded CAMHS hospital, which has seen an increase in patient illness and increasing staff absence due to COVID-19. Informal reports from staff and patients suggested multiple mental health benefits from spending time with Rupert. To quantify the impact of an emotional support dog on the unit, it was agreed to perform a service evaluation on mood, communication and anxiety of both patients and staff.

**Methods:**

A questionnaire, using a Likert-type rating scale, was given to staff and patients before and after spending time with Rupert. Questions asked for ratings of mood, anxiety, and comfort in communicating on a scale from ‘very low’ to ‘very high’. The data collection took place in the last 3 months of 2021. In all, 19 people completed the questionnaire. Because of the small sample size, non-parametric bootstrap resampling methods were used to test before-and-after paired differences for individual participants.

**Results:**

Because the rating scale is ordinal, care needs to be exercised in interpreting differences, but in broad terms a unit increase is equivalent to an improvement, for example, from ‘low’ to ‘neutral’. On average, patients reported statistically significant improvements in mood (mean diff: 1.14, 95% CI: [0.43, 1.71]), anxiety (mean diff: 2.00, 95% CI: [1.43, 2.57]), and communication (mean diff: 1.00, 95% CI: [0.43, 1.86]). Results for staff were similar with improvements in mood (mean diff: 1.08, 95% CI: [0.83, 1.58]) and anxiety (mean diff: 0.83, 95% CI: [0.50, 1.25]) but smaller in communication (mean diff: 0.33, 95% CI: [0.08, 0.67]).

**Conclusion:**

Taking an emotional support dog into a CAMHS Hospital produced clear benefits, with consistently positive feedback from sessions and no negative effects. Such was the improvement in both patient and staff well-being, staff have since been encouraged to register their dogs too.

